# Maternal vitamin D, cord blood cytokines, and early childhood allergic diseases: a 2-year cohort study

**DOI:** 10.1016/j.jped.2026.101544

**Published:** 2026-04-10

**Authors:** Zheng Zhang, Tao Zhan, Jiahui Hu, Yongxing Zhong

**Affiliations:** a Shaoxing Maternity and Child Health Care Hospital, Department of Pediatrics, Shaoxing, China; b Shaoxing Maternity and Child Health Care Hospital, Department of Obstetrics and Gynecology, Shaoxing, China

**Keywords:** Vitamin D, Cytokines, Allergic diseases, Cohort study, Infant

## Abstract

**Objective:**

Maternal prenatal vitamin D is linked to offspring’s disease risk, but its specific association with offspring’s immune development is understudied. This study aimed to explore the relationships between maternal prenatal vitamin D, umbilical cord blood cytokines, and offspring’s allergic diseases within 2 years of life.

**Methods:**

Term infants born in 2023 at Shaoxing Maternal and Child Health Care Hospital (China) were included. Ten cord blood cytokines were quantified by flow cytometry. Logistic regression evaluated maternal vitamin d-child allergy and cytokine-allergy associations, with linear regression for vitamin d-cytokine relationships.

**Results:**

After 2 years of follow-up, maternal prenatal vitamin D deficiency was linked to higher allergic disease risk in offspring (odds ratio = 1.71, 95% confidence interval:1.03–2.87), and maternal vitamin D levels correlated positively with cord blood interleukin-10 (*r* = 0.33, *P* < 0.001). After confounder adjustment, cord blood interleukin-4 (odds ratio = 2.38) and interleukin-10 (odds ratio = 0.78) emerged as independent risk and protective factors for childhood allergic diseases, respectively.

**Conclusions:**

Normal maternal prenatal vitamin D status is tied to lower allergic disease risk in offspring, with umbilical cord blood cytokines (especially interleukin-4 and interleukin-10) potentially mediating this relationship in early childhood.

## Introduction

Childhood allergic diseases are chronic inflammatory disorders that often manifest in early life and impose a substantial burden on pediatric health worldwide and the long-term quality of life of affected children. Accumulating evidence indicates that both genetic and environmental factors contribute to their development and severity, with perinatal exposures playing a critical role [1,2]. Vitamin D, primarily synthesized in the skin upon sunlight exposure, has been implicated in various pathological conditions, including infections and allergic diseases, due to its deficiency [3]. A growing body of research highlights the importance of vitamin D in the pathogenesis of allergic diseases, and with the rising global prevalence of allergic disorders, the relationship between prenatal vitamin D status and childhood allergic diseases has garnered increasing clinical and research attention.

Given that the fetal immune system is highly plastic and uniquely sensitive to maternal nutritional cues during pregnancy, clarifying how maternal prenatal vitamin D modulates fetal immune development via regulating umbilical cord blood cytokine profiles and further impacts early childhood allergic disease risk is critical for identifying modifiable preventive targets and developing early intervention strategies for pediatric allergic diseases.

Vitamin D exerts immunomodulatory effects by influencing the Th1/Th2 and Th17/Treg cell balance, natural killer cell activity, and cytokine production. To determine whether vitamin D deficiency increases the risk of allergic diseases and to elucidate the potential immunological mechanisms, the authors hypothesized that vitamin D deficiency during pregnancy alters umbilical cord blood cytokine profiles, which in turn contribute to the development of allergic diseases in children aged 0–2 years. This study thus investigated the associations among maternal prenatal vitamin D levels, cord blood cytokine profiles, and early childhood allergic disease risk, aiming to provide empirical evidence for the perinatal nutritional regulation of pediatric allergic diseases.

## Materials and methods

### Study population

This prospective cohort study recruited 500 pregnant women from Shaoxing Women and Children's Hospital in 2023. All participants provided written informed consent after the detailed study procedures, potential risks, and benefits were explained. The study was approved by the Ethics Committee of Shaoxing Maternal and Child Health Hospital (Approval no 2023015).

Inclusion criteria were: ① age ≥ 18 years; ② singleton pregnancy; ③ complete clinical and follow-up data. Exclusion criteria for pregnant women included: ① cardiac, hepatic, or renal dysfunction; ② history of malignant tumors; ③ acute or chronic infections; ④ intention to terminate pregnancy. Neonates who developed respiratory distress syndrome or pathological jaundice were also excluded.

Among the 500 initially recruited mother-infant pairs, 195 were excluded (82 due to incomplete clinical/follow-up data, 45 due to maternal organ dysfunction/infections, 38 due to neonatal respiratory distress syndrome/pathological jaundice, 30 due to maternal intention of pregnancy termination). No significant differences in baseline characteristics (maternal age, pre-pregnancy BMI, education level, gestational age, infant sex, and birth weight) were observed between excluded and included dyads (all *P* > 0.05; data shown in Supplemental Table 1).

**Fig. 1:**
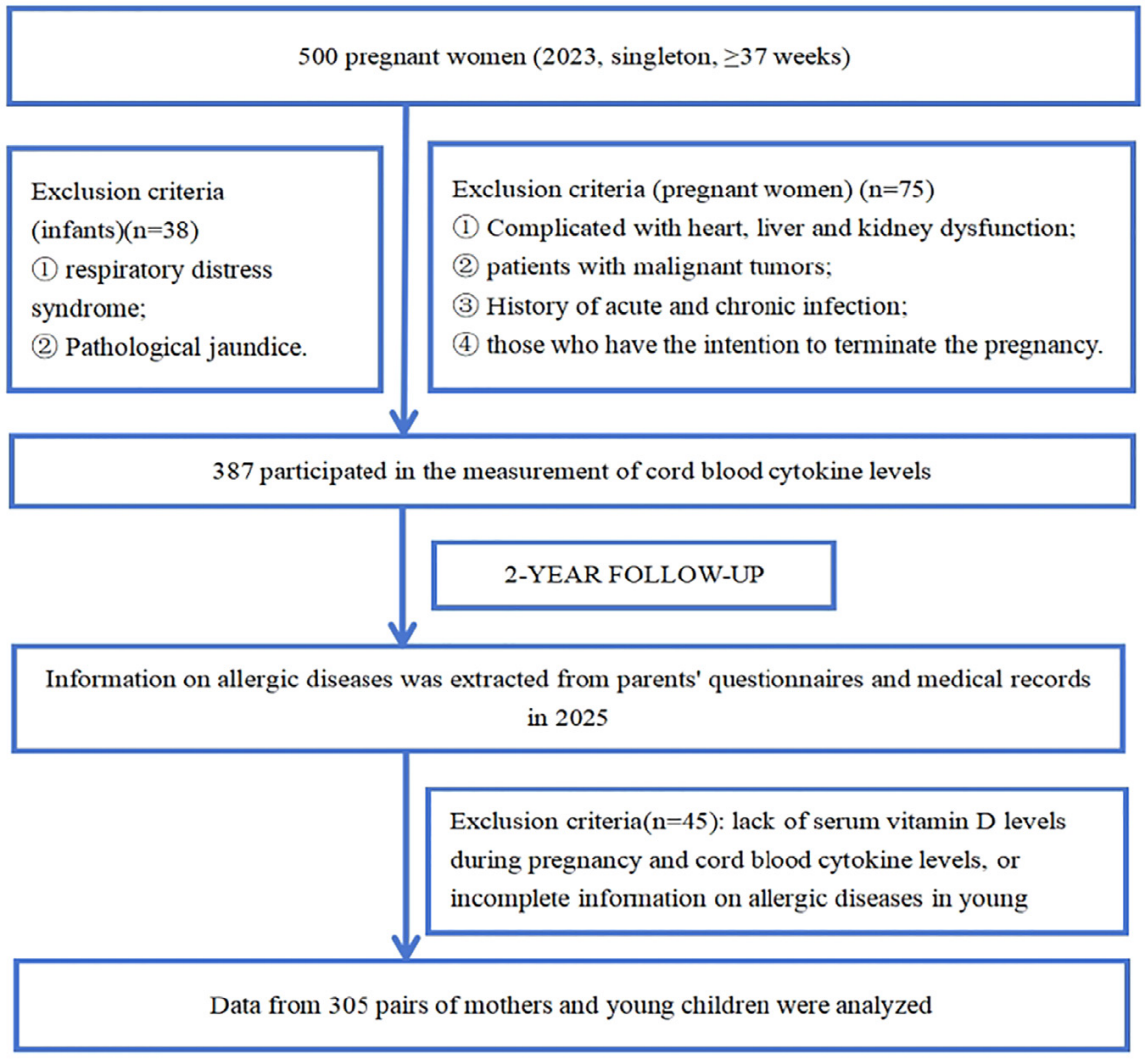
Flow chart of study subject inclusion.

Ultimately, 305 mother-infant pairs were included in the final analysis. A flowchart of participant selection is presented in Figure 1, with the number of excluded subjects for each criterion clearly indicated.

### Clinical data collection

Data were collected using a standardized, investigator-designed questionnaire and electronic medical records:

Maternal data: Lifestyle habits and environmental exposures during pregnancy; demographic characteristics (age, pre-pregnancy BMI (kg/m²), education level); obstetric history (gravidity, parity, gestational age); maternal allergic comorbidities (food allergy, atopic dermatitis, allergic rhinoconjunctivitis) and non-allergic pregnancy complications (hypertensive disorders, gestational diabetes mellitus).

Offspring data: Parent-completed questionnaires and medical records were obtained at 6 months, 1 year, and 2 years of age for longitudinal follow-up of allergic disease trajectories. Information on allergic diseases (food allergy [to milk protein, egg, fish, nuts, peanuts, cashew, sesame, pea, soy, cereal, stone fruits, citrus fruits], atopic dermatitis diagnosed by the Hanifin-Rajka criteria, allergic rhinitis), family history of atopy (atopic dermatitis, allergic rhinitis, asthma, food allergy in first-degree relatives), and allergic reactions in the offspring was collected.

The primary outcome was the development of any allergic disease in offspring at 2 years of age, defined as the presence of at least one physician-diagnosed allergic disease (atopic dermatitis/allergic rhinitis) or confirmed food allergy by 2 years of age, with follow-up data at 6 months and 1 year used to track the onset and progression of allergic manifestations.

### Specimen collection and detection

#### Vitamin D measurement

Maternal fasting peripheral venous blood (5 mL) was collected in the third trimester (28–32 weeks of gestation) in the morning. Serum was separated by centrifugation at 3500 rpm for 10 min and stored at −20 °C until analysis. Serum 25-hydroxyvitamin D [25(OH)D] concentration was measured using high-performance liquid chromatography-tandem mass spectrometry (LC-MS/MS) with the Meiccome 25-Hydroxyvitamin D Detection Kit, following the manufacturer's instructions.

All samples with abnormal 25(OH)D levels (extremely low/high values outside the normal reference range) were re-measured in duplicate to exclude laboratory testing failure, and consistent results were used for final analysis.

#### Cord blood collection

After delivery, 4–6 mL of cord blood was collected from a clamped segment of the umbilical cord using a syringe. Serum was separated within 6–24 h and stored at −70 °C for subsequent cytokine detection.

#### Cytokine measurement

Cytokines, including interleukin-2(IL-2), interleukin-4(IL-4), interleukin-10(IL-10), interleukin-17A(IL-17A), Interferon-γ(IFN-γ) in cord blood serum were quantified using a flow cytometric bead array (CBA) kit (BD Biosciences) on a FACSCalibur flow cytometer (BD Biosciences, USA) in strict accordance with the kit and instrument operating protocols.

### Grouping criteria

Based on serum 25(OH)D levels, pregnant women were categorized into two groups, with the cut-off value referenced to the Institute of Medicine (IOM) 2011 clinical practice guidelines for vitamin D assessment and supplementation [4]:

Vitamin D deficiency group: 25(OH)*D* < 20 ng/mL (*n* = 177)

Vitamin D non-deficiency group: 25(OH)*D* ≥ 20 ng/mL (*n* = 128)

### Statistical analysis

A multivariable logistic regression model with interaction terms was used to test effect modification of vitamin D deficiency on the cytokine-atopy association, with the model constructed as: Outcome (atopy in offspring) ∼ VitD_def (0/1) + ILs (IL-4, IL-10, IL-17A, IFN-γ, IL-2) + VitD_def × ILs + confounders (maternal age, pre-pregnancy BMI, education level, gravidity, parity, delivery mode, maternal allergic comorbidities, gestational hypertension, gestational diabetes mellitus, infant sex, birth weight, family history of atopy in first-degree relatives).

Additionally, supplementary analyses were performed as follows: (1) Univariate and multivariable logistic regression assessed the association between prenatal vitamin D status and allergic diseases after adjusting for potential confounders; (2) Linear regression evaluated the relationship between continuous vitamin D levels and cord blood cytokine levels; (3) Logistic regression examined the independent association between cytokine levels and allergic diseases after confounder adjustment.

pt?>Normally distributed continuous data are presented as mean ± standard deviation (SD); non-normally distributed data as median (interquartile range, IQR); categorical data as n (%). Group comparisons were made using the chi-square test or Fisher’s exact test for categorical data, and independent samples *t*-test or Mann-Whitney U test for continuous data, as appropriate. All analyses were performed using IBM SPSS Statistics (Version 29.0) and GraphPad Prism (Version 10.0). A two-tailed P-value < 0.05 was considered statistically significant.

## Results

### Baseline characteristics

A total of 305 mother-infant pairs completed the 2-year follow-up. The mean maternal age was 29 years. Baseline characteristics are summarized in Table 1. The term "Characteristic dermatitis" was revised to Atopic dermatitis for accurate clinical classification. As expected, mean 25(OH)D levels significantly differed between the deficiency (16.32 ± 2.43 ng/mL) and non-deficiency (24.37 ± 3.67 ng/mL) groups (*P* < 0.001). No other significant differences in baseline characteristics were observed between the two groups (all *P* > 0.05), indicating good group balance.

**Table 1:** Demographic characteristics of mothers and young children.

Characteristic	Vitamin D deficiency group (*n* = 177)	Vitamin D non-deficiency group (*n* = 128)	χ^2/^t/Z	*P*
Age (years)	29.76 ± 3.77	29.84 ± 4.10	0.16	0.87
Vitamin D levels (ng/mL)	16.32 ± 2.43	24.37 ± 3.67	23.05	<0.001
Pre-pregnancy overweight/obese, n (%)	7(3.9)	9(7.0)	1.39	0.24
Education level, n (%)			5.93	0.12
Junior high school and below	14(7.9)	11(8.6)		
High school	52(29.4)	39(30.5)		
College and undergraduate	98(55.4)	69(53.9)		
Graduate student or above	13(7.3)	9(7.0)		
Gravidity (≥2 times), n (%)	86(48.6)	68(53.1)	0.61	0.43
Parity (≥2 times)	56 (31.6)	49 (38.3)	1.45	0.23
Mode of delivery, n (%)			0.02	0.88
Vaginal delivery	97 (54.8)	69 (53.9)		
Cesarean section	80 (45.2)	59 (46.1)		
Maternal atopic dermatitis/eczema, n(%)	23 (13.0)	23 (18.0)	1.44	0.23
Maternal asthma, n (%)	1 (0.6)	0	1.39	0.24
Maternal food allergy, n (%)	5 (2.8)	3 (2.3)	0.07	0.79
Gestational hypertension, n (%)	10 (5.6)	5 (3.9)	0.50	0.48
Gestational diabetes mellitus, n (%)	28 (15.8)	19 (14.8)	0.06	0.80
Family history of atopy, n (%)	42 (23.7)	28 (21.9)	0.14	0.71
Infant sex, n (%)			0.71	0.40
Male	104 (58.8)	69 (53.9)		
female	73 (41.2)	59 (46.1)		
Birth weight (kg)	3.40 ± 0.41	3.31 ± 0.51	−1.76	0.08

As stated in the Study Population section, no significant differences in baseline characteristics were found between excluded and included mother-infant dyads (all *P* > 0.05; Supplemental Table 1).

### Association between maternal vitamin D status and allergic diseases in offspring

Maternal prenatal vitamin D deficiency was associated with a significantly higher risk of allergic diseases in offspring at 2 years of age [odds ratio (OR) = 1.71, 95%confidence interval (CI): 1.03–2.87; *P* = 0.04] (See Table 2 for details), even after adjusting for maternal age, pre-pregnancy BMI, education level and infant sex/birth weight.

**Table 2:** Association between vitamin D nutritional status during pregnancy and allergic diseases in children.

	Allergic diseases in children			
	β	SE	OR (95%CI)	P
Vitamin D deficiency group (*n* = 177)	0.54	0.26	1.71(1.03–2.87)	0.04
Vitamin D non-deficiency group (*n* = 128)			1.00	

### Association between maternal vitamin D status and cord blood cytokines

**Fig. 2:**
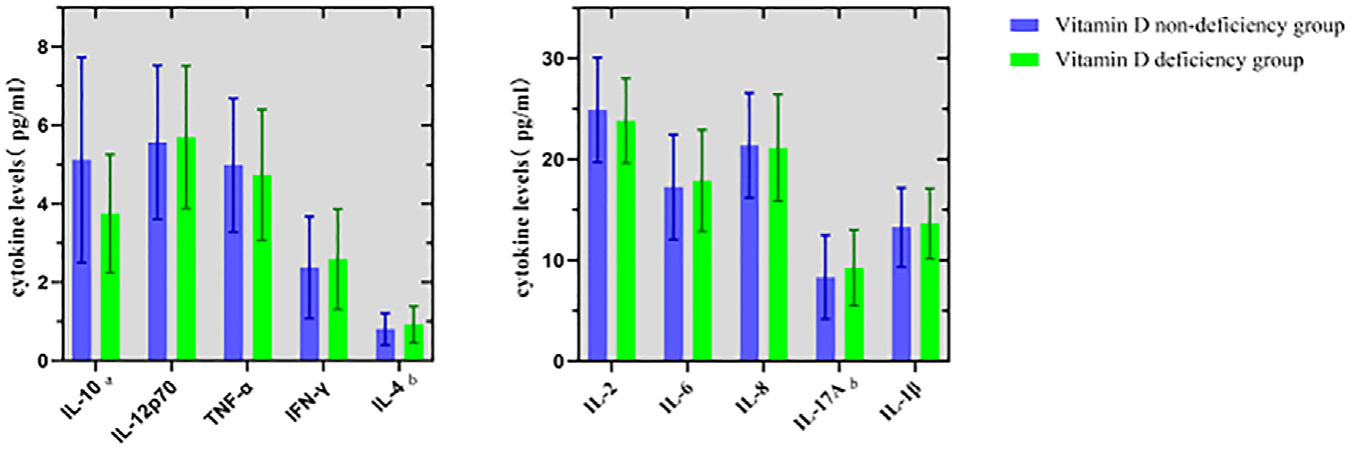
Comparison of cord blood cytokine levels between vitamin D deficiency and non-deficiency groups.

**Fig. 3:**
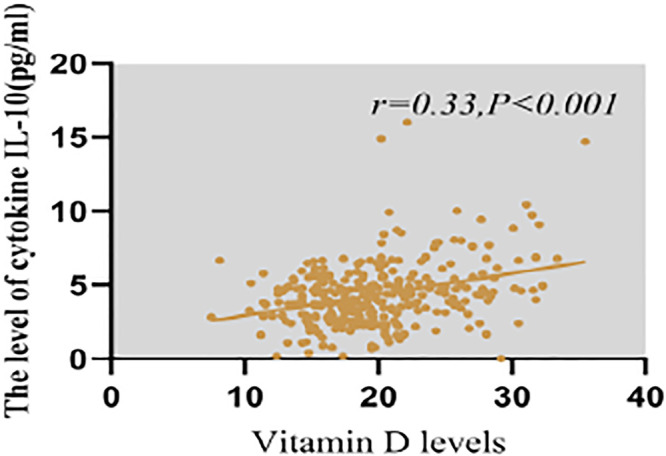
Correlation between maternal 25(OH)D levels and cord blood IL-10 levels.

Levels of IL-4, IL-10, and IL-17A in cord blood serum significantly differed between the vitamin D deficiency and non-deficiency groups (all *P* < 0.05, Figure 2). Linear regression analysis revealed a significant positive correlation between maternal 25(OH)D levels and cord blood IL-10 (*r* = 0.33, *P* < 0.001, Figure 3), with no significant correlations found between vitamin D levels and other cytokines (all *P* > 0.05). The interaction analysis showed that vitamin D deficiency modified the association between IL-4 and offspring atopy (P for interaction = 0.03), while no significant interaction was observed for IL-10 (P for interaction = 0.12).

### Association between cord blood cytokines and allergic diseases

**Fig. 4:**
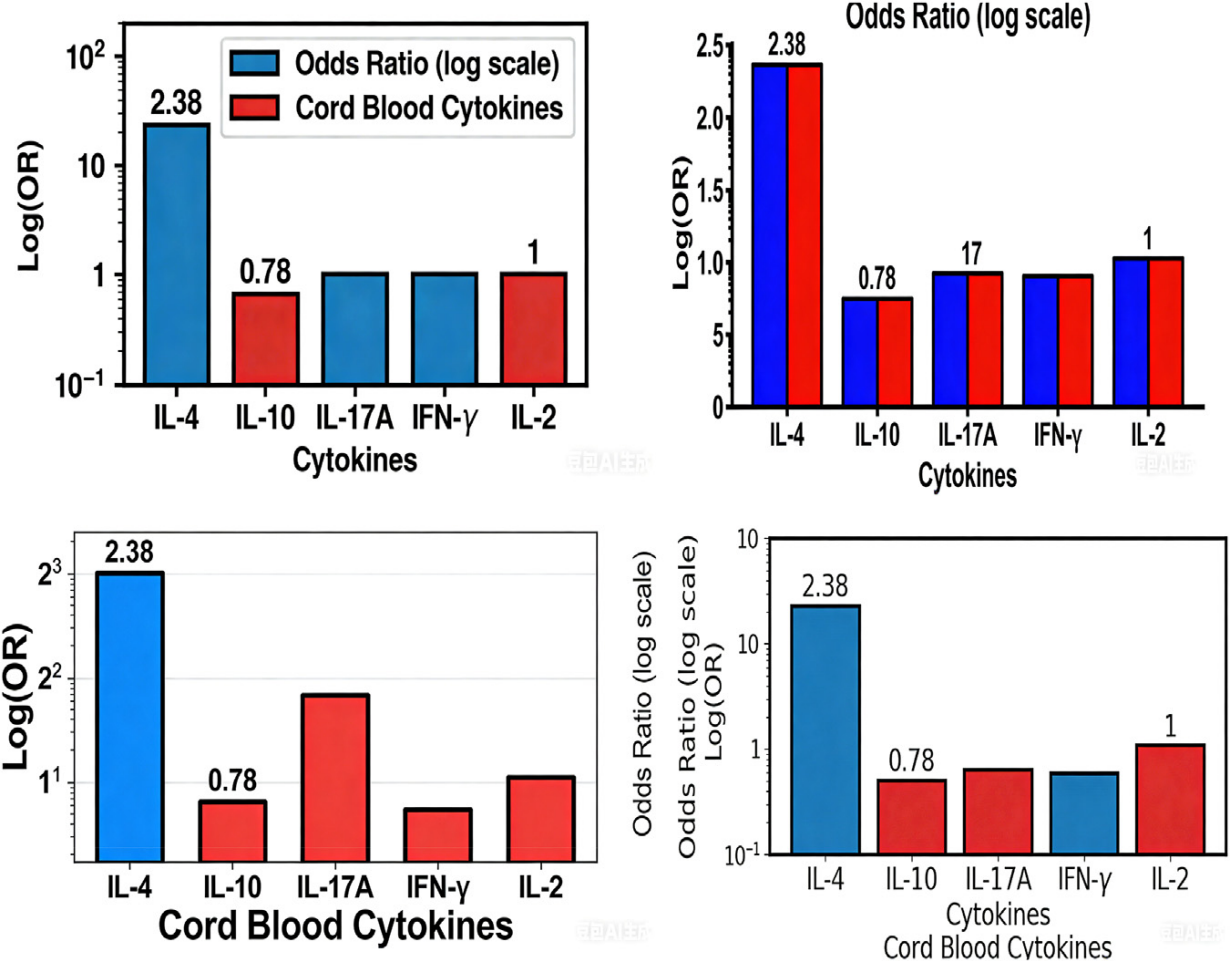
Forest plot of cord blood cytokines in relation to childhood allergic diseases (logarithmic Y-axis scale).

After adjusting for potential confounders (maternal age, pre-pregnancy overweight/obesity, education level, gravidity, parity, delivery mode, maternal allergic comorbidities (food allergy, atopic dermatitis, allergic rhinoconjunctivitis), maternal asthma, gestational hypertension, gestational diabetes mellitus, infant sex, birth weight, family history of atopy in first-degree relatives), logistic regression analysis showed that cord blood IL-4 was an independent risk factor and IL-10 an independent protective factor for allergic diseases in offspring. Each unit increase in IL-4 was associated with an OR of 2.38 (95% CI: 1.31–4.33). For IL-10, each unit increase was associated with an OR of 0.78 (95% CI: 0.66–0.91), equivalent to a 22% reduced risk per unit increase (Figure 4).

Figure 4 uses a logarithmic scale for the Y-axis (OR values) to improve the visibility and interpretability of the association results.

## Discussion

Calcitriol, the active form of vitamin D, mediates its biological effects via the vitamin D receptor (VDR) expressed in a variety of immune cells, including T cells, B cells, dendritic cells and natural killer cells. Low vitamin D levels have been associated with increased allergy and asthma severity [5], and recent cross-sectional research has further linked prenatal vitamin D deficiency to early allergic rhinitis in neonates [6], supporting a critical role of maternal vitamin D in fetal allergic susceptibility. This study investigated the associations among maternal vitamin D status, cord blood cytokines, and their impact on allergic disease development in offspring within 2 years of age, with the main findings being: (1) Maternal vitamin D levels are positively correlated with cord blood IL-10 levels; (2) Normal maternal prenatal vitamin D levels are associated with a reduced risk of allergic diseases in offspring; (3) Vitamin D deficiency modifies the IL-4-atopy association, and cord blood cytokines (IL-4, IL-10) may partially mediate the relationship between maternal vitamin D and offspring allergic disease risk.

Our finding of an inverse correlation between maternal vitamin D and offspring allergy risk is consistent with prior studies [7,8], including those linking low maternal vitamin D intake to increased risks of wheezing, asthma, eczema, and sensitization in young children [9,10]. A prospective cohort study also suggested that maternal fish oil supplementation (a source of vitamin D) may protect against childhood asthma [10], supporting the role of vitamin D in modulating fetal and early postnatal inflammatory pathways [11,12]. The consistency of the present results with cross-cultural studies (e.g., the Greek neonate study by Kokkinari et al. [6]) further validates the universal role of prenatal vitamin D in early childhood allergic disease susceptibility, regardless of geographical and ethnic differences.

The present study provides new evidence on how prenatal vitamin D influences fetal immune programming, a key window for the development of allergic diathesis. Vitamin D enhances regulatory T (Treg) cell function [13] and promotes IL-10 secretion by B cells, Tregs, and dendritic cells [[14], [15], [16]]. The positive correlation between vitamin D and cord blood IL-10 observed in our cohort suggests that vitamin D can modulate the fetal immune cytokine milieu in utero, by upregulating the production of anti-inflammatory cytokines and thus shaping a more tolerogenic fetal immune microenvironment. This is a critical finding because the fetal immune system is in a state of immune tolerance, and disruptions to this balance by low vitamin D and subsequent reduced IL-10 production may prime the offspring for an exaggerated Th2 response after birth upon allergen exposure.

The cytokine microenvironment in the perinatal period is a key determinant of allergic disease pathogenesis [17,18], and atopic dermatitis, as the most common early-onset allergic disease, is particularly closely linked to the imbalance of pro-inflammatory and anti-inflammatory cytokines in cord blood. IL-10 has potent immunosuppressive and anti-inflammatory properties [19,20]; for instance, IL-10 deficiency in mice leads to enhanced allergic airway inflammation [21], and clinical studies have shown that reduced cord blood IL-10 levels are associated with an increased risk of early-onset atopic dermatitis in infants. The identification of IL-10 as a protective factor for early childhood allergic diseases aligns with numerous studies [22,23], further confirming that IL-10-mediated immune tolerance is a key protective mechanism against pediatric allergic diseases.

Conversely, IL-4, a key Th2 cytokine, drives IgE class switching [[24], [25], [26]] and is a central regulator of allergen-specific Th2 responses [27,28]; our finding that vitamin D deficiency modifies the IL-4-atopy association indicates that low maternal vitamin D may amplify the pro-allergic effect of IL-4, which together leads to a significantly increased risk of allergic diseases in offspring. The confirmation of IL-4 as an independent risk factor reinforces its established and central role in the initiation and development of early childhood allergic diseases, especially atopic dermatitis and food allergy.

The mechanistic interplay between maternal vitamin D, cord blood cytokines, and early childhood allergic diseases can be summarized as follows: maternal prenatal vitamin D deficiency downregulates the secretion of anti-inflammatory cytokine IL-10 in cord blood and amplifies the pro-allergic effect of IL-4, which disrupts the fetal Th1/Th2 immune balance and impairs immune tolerance development. This in utero immune programming leads to an increased susceptibility to allergen exposure after birth, ultimately increasing the risk of allergic disease onset in the first 2 years of life.

For atopic dermatitis, the most common early allergic manifestation in the present study cohort, this mechanistic pathway is particularly relevant: reduced cord blood IL-10 impairs the skin barrier function and immune tolerance of the offspring, while elevated IL-4 promotes Th2 polarization and IgE production, which together trigger the onset of atopic dermatitis and further increase the risk of subsequent allergic march (e.g., progression to allergic rhinitis and asthma).

In summary, this study systematically explores the effects of maternal vitamin D status on cord blood cytokine profiles and early-life allergic disease risk in offspring, and for the first time demonstrates the effect modification of vitamin D deficiency on the IL-4-atopy association in a Chinese cohort. These findings highlight the importance of maternal prenatal vitamin D supplementation as a potential preventive strategy for early childhood allergic diseases.

## Limitations

This study has several limitations. First, it does not provide direct evidence of cytokine mediation, and causal inferences cannot be made due to the observational study design. Second, the sample size was relatively small, the study was conducted at a single center, and the follow-up period was short (2 years), which may limit the generalizability of the results and the ability to assess the long-term impact on allergic disease progression (e.g., allergic march).

Third, allergic disease diagnosis for some mild cases was based on parent-completed questionnaires, which may lead to potential classification error and information bias.

Fourth, only a limited number of cytokines were measured, and other immune factors (e.g., chemokines, growth factors) that may be involved in the vitamin d-allergy pathway were not assessed. Future multi-center prospective cohort studies with larger sample sizes, longer follow-up periods, and comprehensive immune factor detection are needed to confirm these findings and explore the underlying causal mechanisms. Additionally, interventional studies on maternal prenatal vitamin D supplementation are warranted to validate the preventive effect on early childhood allergic diseases.

## Conclusions

Normal maternal prenatal vitamin D status was positively correlated with cord blood IL-10 levels in offspring. Cord blood IL-4 and IL-10 were identified as independent risk and protective factors, respectively, for allergic diseases at 2 years of age, and vitamin D deficiency modified the pro-allergic effect of IL-4. Although a direct mediating role of cytokines was not established, these findings offer novel insights into the immunological mechanisms underlying the relationships between maternal vitamin D, cord blood immunity, and offspring allergy risk, and highlight that maternal prenatal vitamin D status is a key modifiable factor for early childhood allergic disease prevention, warranting further clinical and translational investigation.

## Funding

This study was supported by the Shaoxing Health Science and Technology Plan Project (Grants no 2022KY039, 2023SKY049)

## Conflicts of interest

The authors declare no conflicts of interest.

## Data Availability

Data are available within the article or its supplementary materials.

## Appendix Supplementary materials

Image, application 1
