# Supplementary material for: Maternal vitamin D, cord blood cytokines, and early childhood allergic diseases: a 2-year cohort study
Source: J Pediatr (Rio J). 2026 Apr 10;102(3):101544. doi: 10.1016/j.jped.2026.101544 (PMC13091998; doi:10.1016/j.jped.2026.101544)
Supplement: Supplementary file 1 [file mmc1.doc]

**JPED-D-25-00539_ Supplementary mATERIAL**

**Supplementary Table S1** Demographic characteristics of mothers and young children.

| **Characteristic** | Included (n = 305) | Excluded (n = 195) | χ^2/^t/Z | *P* |
| --- | --- | --- | --- | --- |
| Age (years) | 29.79 ± 3.90 | 29.91 ± 3.97 | -0.33 | 0.74 |
| Vitamin D levels（ng/mL） | 19.70 ± 4.97 | 18.90 ± 4.68 | 1.79 | 0.07 |
| Pre-pregnancy overweight/obese, n (%) | 16(5) | 16（8.2） | 1.71 | 0.19 |
| Education level, n (%) |  |  | 1.03 | 0.30 |
| Junior high school and below | 25(8.2) | 14(7.1) |  | |
| High school | 106(34.8) | 54(27.7） |  | |
| College and undergraduate | 156(51.1) | 121(62.1) |  | |
| Graduate student or above | 18(5.9) | 6(3.0） |  | |
| Gravidity (≥2 times), n (%) | 154(50.5) | 95(48.7) | 0.15 | 0.70 |
| Parity (≥2 times) | 105 (34.4) | 62 (31.8) | 0.37 | 0.4 |
| Mode of delivery, n (%) |  |  | 0.01 | 0.92 |
| Vaginal delivery | 166 (54.4) | 107 (54.9) |  | |
| Cesarean section | 139 (45.6) | 88 (45.1) |  | |
| Maternal atopic dermatitis/eczema, n(%) | 46 (15.1) | 23 (11.8) | 1.08 | 0.30 |
| Maternal asthma, n (%) | 1 (0.3) | 0 | 0.64 | 0.42 |
| Maternal food allergy, n (%) | 8 (2.6) | 4 (2.1) | 0.17 | 0.68 |
| Gestational hypertension, n (%) | 15 (5.0) | 8 (4.1) | 0.19 | 0.67 |
| Gestational diabetes mellitus, n (%) | 47 (15.4) | 29 (14.9) | 0.03 | 0.86 |
| Family history of atopy, n (%) | 70 (23.0) | 35(17.9) | 1.79 | 0.18 |
| Infant sex, n (%) |  |  | 0.94 | 0.33 |
| Male | 173(56.7) | 102 (52.3) |  | |
| female | 132 (43.3) | 93 (47.7) |  | |
| Birth weight (kg) | 3.36 ± 0.46 | 3.28 ± 0.59 | 1.70 | 0.09 |
